# Supplementary material for: An index of access to essential infrastructure to identify where physical distancing is impossible
Source: Nat Commun. 2022 Jun 14;13:3355. doi: 10.1038/s41467-022-30812-8 (PMC9198068; doi:10.1038/s41467-022-30812-8)
Supplement: Supplementary file 1 — Supplementary Information [file 41467_2022_30812_MOESM1_ESM.pdf]

Supplementary Information

# An index of access to essential infrastructure to identify where physical distancing is impossible

Isabel Günther[1,2], Kenneth Harttgen[1,2,*], Johannes Seiler[3], and Jürg Utzinger[4,5]

[1] Development Economics Group, ETH Zurich, Zurich, Switzerland
[2] NADEL Center for Development and Cooperation, ETH Zurich, Zurich, Switzerland
[3] Department of Statistics, University of Innsbruck, Innsbruck, Austria
[4] Swiss Tropical and Public Health Institute, Allschwil, Switzerland
[5] University of Basel, Basel, Switzerland

April 20, 2022

*E-mail corresponding author: kenneth.harttgen@nadel.ethz.ch

# 1 Supplementary Figures

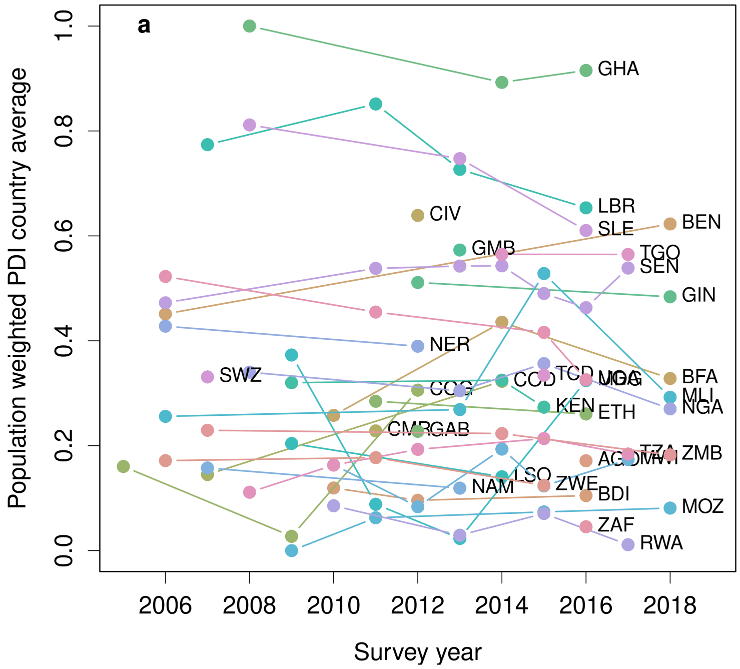


Fig. 1.1: Development of the PDI over time for all countries with complete information. Note that the PDI is normalized between zero and one for the whole time period, accordingly specific values for the index might differ from values in Table 2.1, however this does not change the interpretation of the index; calculations by the authors.

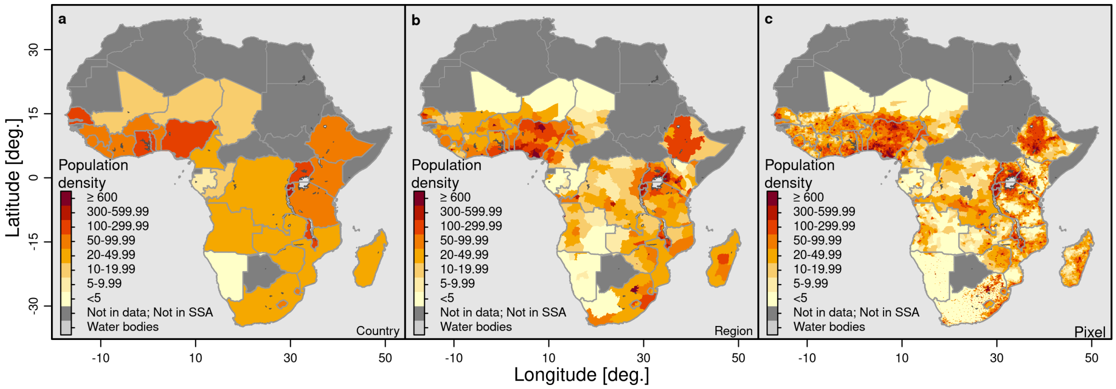


Fig. 1.2: Population density at the country (a), regional (b), and pixel (c) level. Source: Center for International Earth Science Information Network—CIESIN —Columbia University [1]; calculations by the authors.

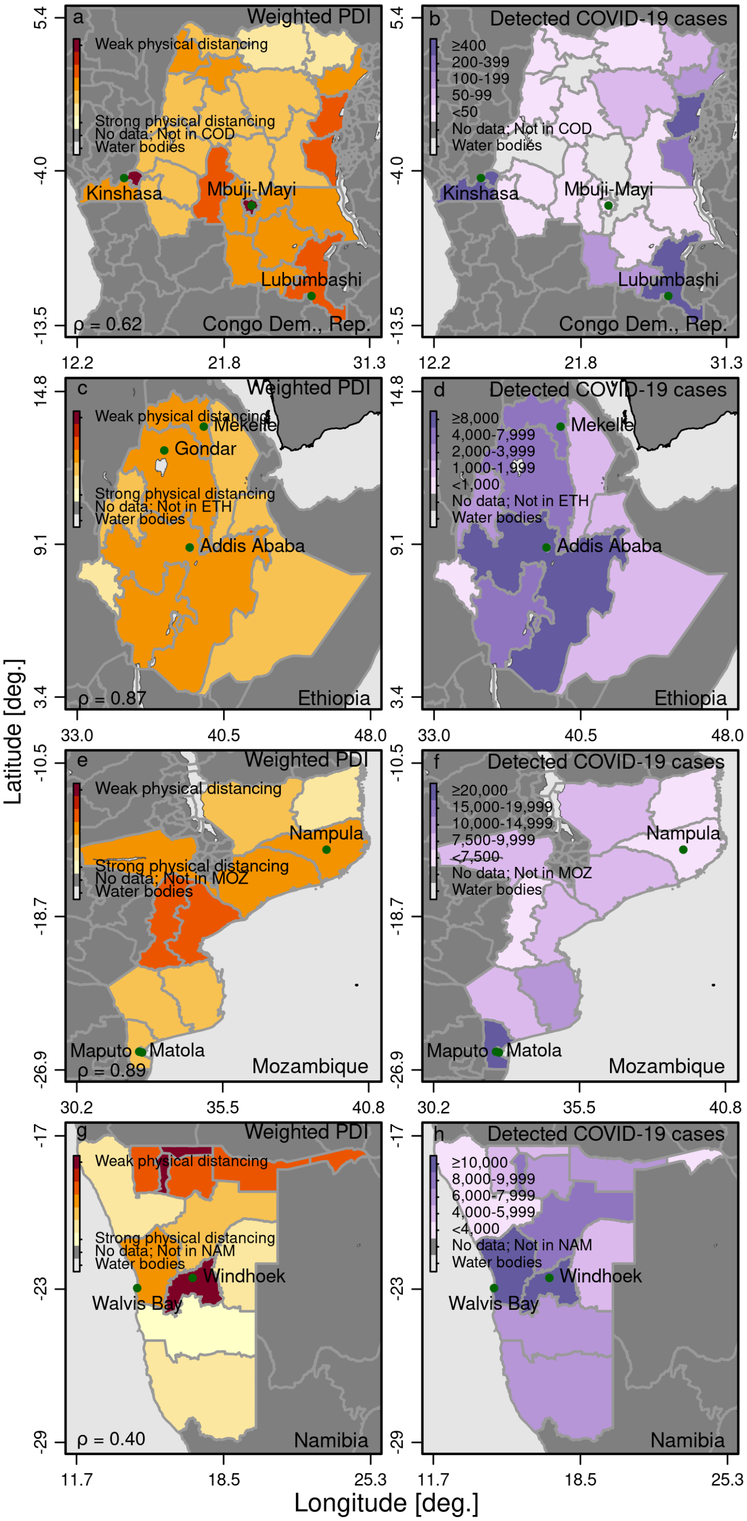


Fig. 1.3: From top to bottom: population weighted PDI (left) and observed cumulative caseload (right) at the regional level for countries with information on the regional caseload. Note that we used the latest available information on the regional caseload, which differs by country. The countries for which information on the regional caseload was publicly available are from top to bottom: Democratic Republic of the Congo; Ethiopia; Mozambique; and Namibia. Source: DHS and several country specific publicly available data sources which we are happy to share upon request; calculations by the authors.

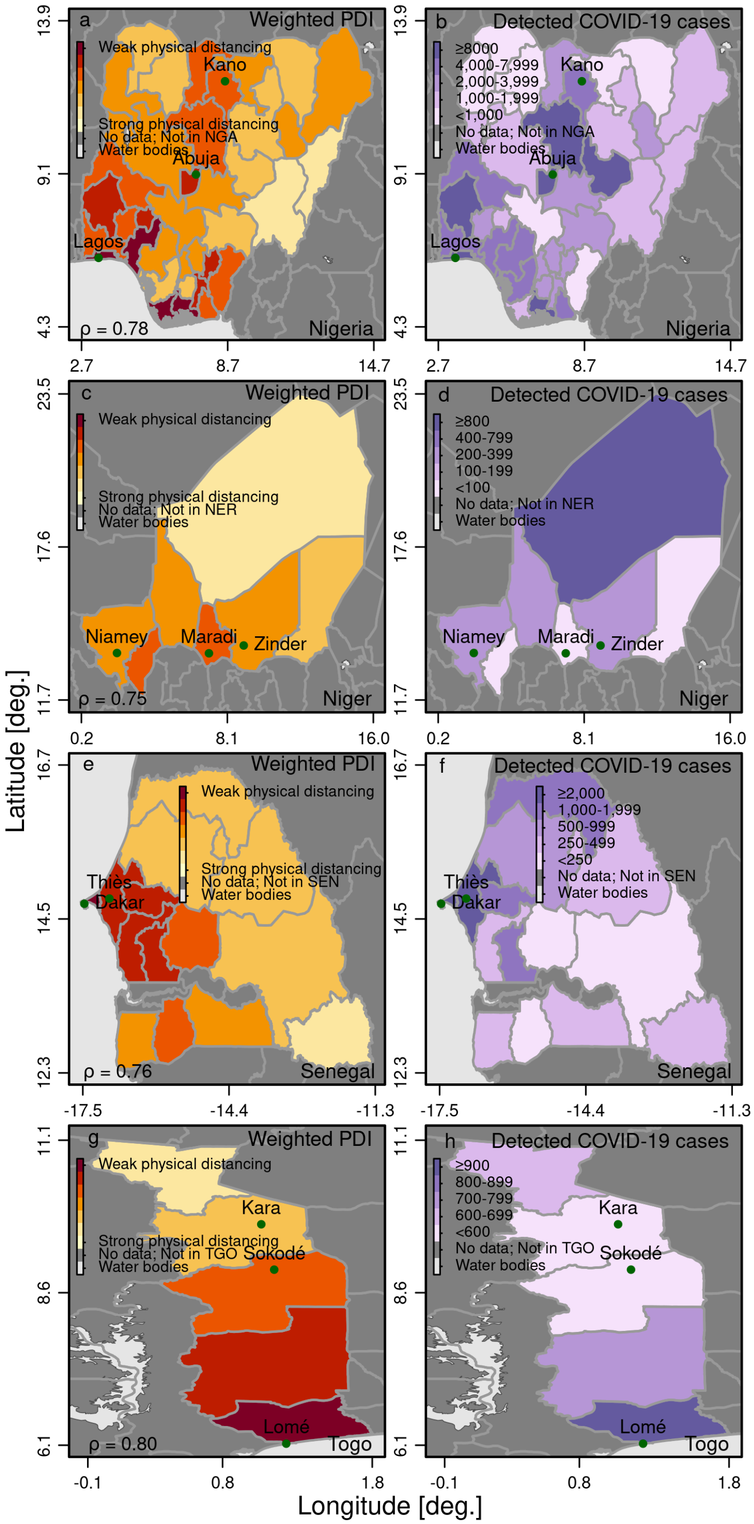


Fig. 1.4: From top to bottom: population weighted PDI (left) and observed cumulative caseload (right) at the regional level for countries with information on the regional caseload. Note that we used the latest available information on the regional caseload, which differs by country. The countries for which information on the regional caseload was publicly available are from top to bottom: Nigeria; Niger; Senegal; and Togo. Source: DHS and several country specific publicly available data sources which we are happy to share upon request; calculations by the authors.

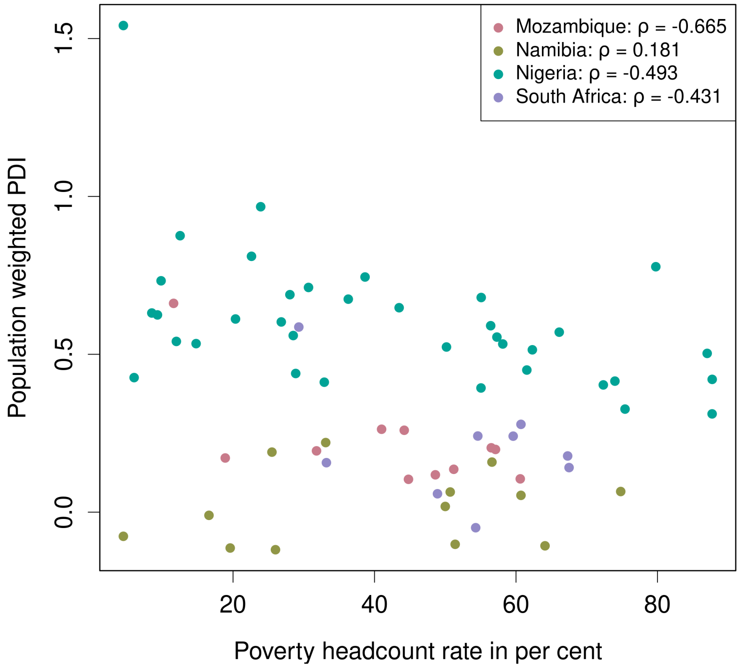


Fig. 1.5: Scatterplot of the population weighted PDI at the regional level and the poverty headcount rate at the regional level for countries where subnational estimates of the poverty headcount rate is available. These countries are Mozambique, Namibia, Nigeria, and South Africa. Source: DHS and National Statistical Offices; calculations by the authors.

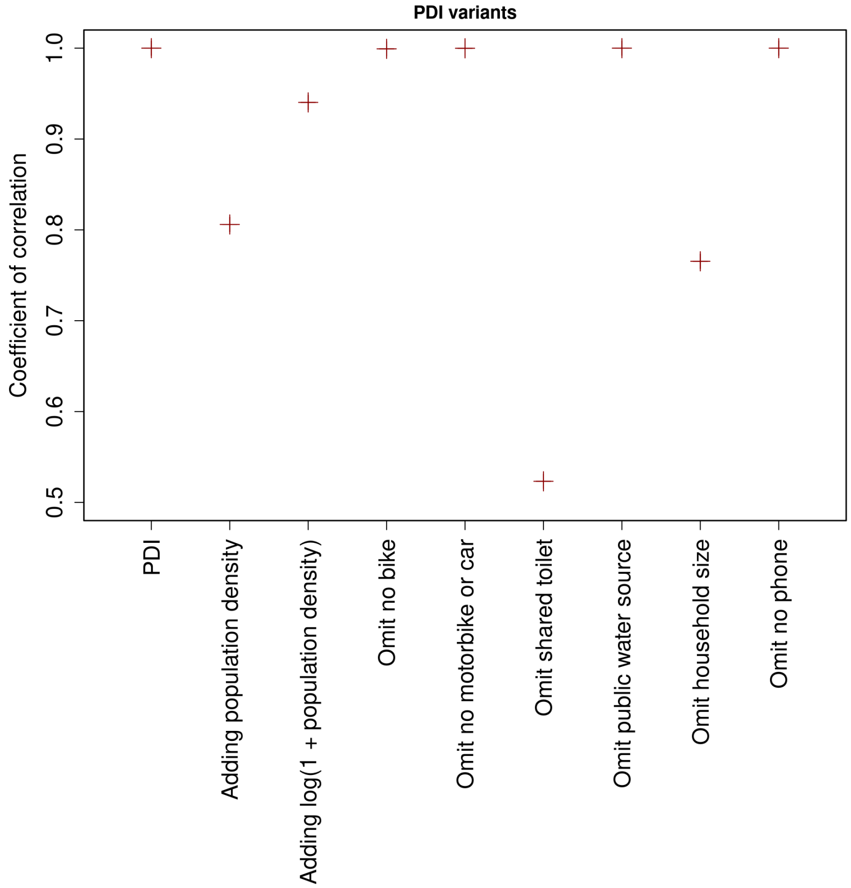


Fig. 1.6: Sensitivity analysis of the index at the country level. Correlation of the unweighted PDI and several variants of the PDI with the population weighted PDI. Source: DHS; calculations by the authors.

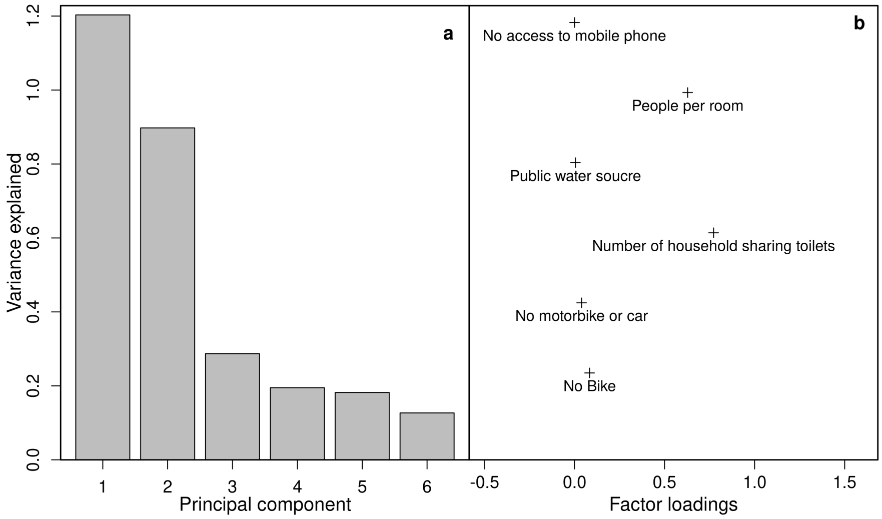


Fig. 1.7: Variance by components of the PCA (a) and factor loadings of the indicators of the PDI by indicator (b). The index includes the following variables: (i) number of households sharing toilet facilities, (ii) usage of public water sources, (iii) persons per room, (iv) without access to ICT, (v) bicycle or other vehicle is not present. Source: DHS; calculations by the authors.

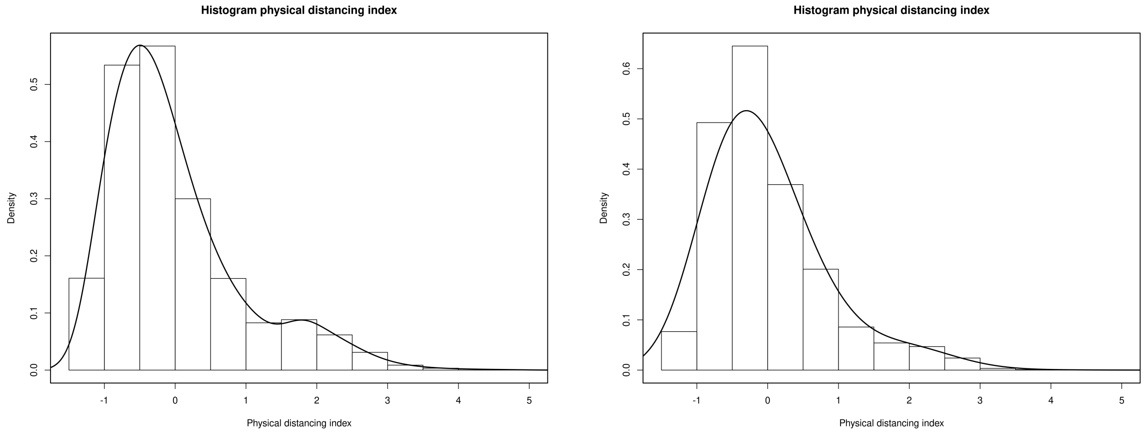


Fig. 1.8: Histogram of the PDI for the complete sample (left) and Madagascar (right) including kernel density estimates. Source: DHS; calculations by authors.

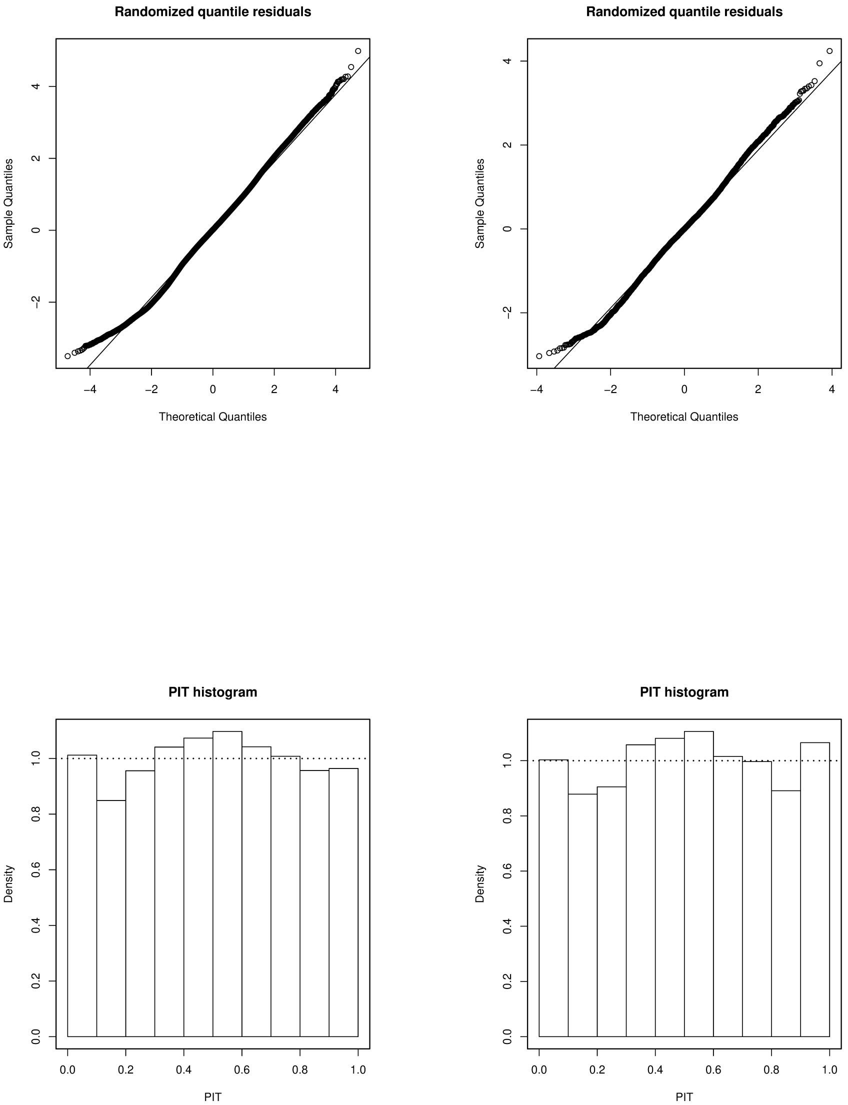


Fig. 1.9: Randomized quantile residuals (top) and PIT-histogram (bottom) of the final model for mainland sub-Saharan Africa (left) and Madagascar (right). Source: DHS; calculations by authors.

# 2 Supplementary Tables

Table 2.1: List of countries with latest available DHS survey year.

| **Country** | **ISO-3 Country code** | **Year** | **Number of households sharing sanitation facility** | **Share of households using public water sources** | **People per room** | **Share of households with no access to mobile phone** | **Share of households without bike or other vehicle** | **Population density (people per km$^2$)** | PDI |
|---|---|---|---|---|---|---|---|---|---|
| Angola | AGO | 2016 | 1.70 | 0.61 | 3.25 | 0.25 | 0.68 | 21 | 0.21 |
| Benin | BEN | 2018 | 4.23 | 0.34 | 3.03 | 0.09 | 0.36 | 99 | 0.75 |
| Burkina Faso | BFA | 2018 | 2.74 | 0.26 | 3.65 | 0.03 | 0.06 | 67 | 0.50 |
| Burundi | BDI | 2016 | 1.39 | 0.90 | 3.03 | 0.52 | 0.77 | 423 | 0.46 |
| Cameroon | CMR | 2011 | 2.14 | 0.53 | 3.15 | 0.29 | 0.69 | 48 | 0.35 |
| Chad | TCD | 2015 | 2.48 | 0.26 | 4.06 | 0.19 | 0.45 | 11 | 0.25 |
| Democratic Republic of the Congo | COD | 2014 | 2.29 | 0.80 | 3.56 | 0.55 | 0.71 | 31 | 0.35 |
| Republic of the Congo | COG | 2012 | 3.36 | 0.48 | 2.87 | 0.15 | 0.87 | 10 | 0.25 |
| Côte d'Ivoire | CIV | 2012 | 4.46 | 0.17 | 3.30 | 0.11 | 0.56 | 69 | 0.75 |
| Ethiopia | ETH | 2016 | 1.99 | 0.61 | 3.14 | 0.37 | 0.95 | 88 | 0.41 |
| Gabon | GAB | 2012 | 2.90 | 0.32 | 2.54 | 0.08 | 0.82 | 7 | 0.14 |
| The Gambia | GMB | 2016 | 2.34 | 0.34 | 5.26 | 0.09 | 0.46 | 174 | 0.83 |
| Ghana | GHA | 2016 | 6.17 | 0.34 | 2.09 | 0.09 | 0.71 | 120 | 1.00 |
| Guinea | GIN | 2018 | 2.84 | 0.26 | 4.03 | 0.08 | 0.52 | 56 | 0.55 |
| Kenya | KEN | 2018 | 3.12 | 0.48 | 2.16 | 0.07 | 0.68 | 79 | 0.45 |
| Lesotho | LSO | 2014 | 2.30 | 0.52 | 2.55 | 0.11 | 0.85 | 63 | 0.35 |
| Liberia | LBR | 2016 | 4.66 | 0.16 | 3.54 | 0.27 | 0.83 | 53 | 0.75 |
| Madagascar | MDG | 2016 | 2.90 | 0.65 | 2.84 | 0.56 | 0.73 | 41 | 0.40 |
| Malawi | MWI | 2017 | 1.74 | 0.21 | 2.84 | 0.48 | 0.61 | 162 | 0.39 |
| Mali | MLI | 2018 | 2.15 | 0.24 | 3.67 | 0.10 | 0.23 | 14 | 0.22 |
| Mozambique | MOZ | 2018 | 1.32 | 0.36 | 2.86 | 0.29 | 0.61 | 36 | 0.18 |
| Namibia | NAM | 2013 | 2.56 | 0.17 | 2.32 | 0.04 | 0.52 | 3 | 0.00 |
| Niger | NER | 2012 | 2.35 | 0.44 | 4.08 | 0.28 | 0.61 | 14 | 0.28 |
| Nigeria | NGA | 2018 | 2.52 | 0.18 | 3.00 | 0.10 | 0.58 | 204 | 0.58 |
| Rwanda | RWA | 2017 | 1.43 | 0.84 | 2.30 | 0.37 | 0.86 | 498 | 0.37 |
| Senegal | SEN | 2017 | 1.92 | 0.18 | 5.87 | 0.03 | 0.78 | 102 | 0.75 |
| Sierra Leone | SLE | 2016 | 3.86 | 0.44 | 3.77 | 0.28 | 0.81 | 82 | 0.76 |
| South Africa | ZAF | 2016 | 2.04 | 0.22 | 1.92 | 0.04 | 0.67 | 43 | 0.20 |
| Eswatini (Swaziland) | SWZ | 2007 | 2.69 | 0.36 | 2.89 | 0.33 | 0.72 | 66 | 0.44 |
| Tanzania | TZA | 2017 | 1.90 | 0.54 | 3.00 | 0.17 | 0.54 | 61 | 0.32 |
| Togo | TGO | 2017 | 4.45 | 0.36 | 2.56 | 0.08 | 0.48 | 138 | 0.80 |
| Uganda | UGA | 2016 | 2.33 | 0.37 | 2.96 | 0.23 | 0.58 | 173 | 0.52 |
| Zambia | ZMB | 2018 | 1.96 | 0.26 | 3.29 | 0.23 | 0.56 | 22 | 0.24 |
| Zimbabwe | ZWE | 2015 | 2.14 | 0.16 | 2.49 | 0.09 | 0.65 | 40 | 0.26 |

The table reports the country averages of the indicators of the PDI. Estimates are calculated using the DHS survey weights. Source: DHS; calculations by the authors.

Table 2.2: Model comparison of the estimated models.

| **Model/distribution** | **Sub-Saharan Africa** | | | **Madagascar** | | |
|---|---|---|---|---|---|---|
| | **DIC** | **WAIC 1** | **WAIC 2** | **DIC** | **WAIC 1** | **WAIC 2** |
| NO | 1,140,500 | 1,140,501 | 1,140,501 | 27,639 | 27,648 | 27,650 |
| NO | 1,076,096 | 1,076,213 | 1,076,217 | 26,380 | 26,417 | 26,440 |
| TF | 1,038,941 | 1,038,963 | 1,038,966 | 25,839 | 25,846 | 25,852 |
| SN2 | **1,031,569** | **1,031,603** | **1,031,603** | 23,946 | 23,955 | 23,978 |
| ST2* | 956,1161 | 956,121 | 956,122 | **23,780** | **23,770** | **23,785** |

Source: DHS; calculations by the authors.

The normal distribution has been modeled once using covariats only in $\mu$ and once covariats in $\mu$ and $\sigma$.
The model indicated with an asterisk did not converge, and accordingly we did not use it to predict the PDI.

# 3 Methodological details

## 3.1 Construction of the PDI

The geo-spatial estimation is based on the one-dimensional PDI that is based on factors associated with the household's infrastructure. This variable is constructed using a PCA approach similar to the one proposed by Filmer and Pritchett [2] for the construction of a household's asset index, which reduces the dimensions effectively. The main idea of the construction of the PDI is to construct at the household level an aggregated one-dimensional index over the range of different variables of household infrastructure. These variables represent the household's lack of important infrastructure concerning the risk of the spread of diseases:

$$A_i = b_1 a_{i1} b_2 a_{i2} + ... + b_p a_{ip} \tag{1}$$

$$a_{ip} = ß_p c_i + u_{ip} \tag{2}$$

for $i = 1, \ldots, N$ households and $p = 1, \ldots, P$ infrastructural variables. $A_i$ is the PDI, and $a_{i1}$ refers to the respective infrastructural variable of household $i$ recorded in the DHS data, and the $b_p$ are the weights for each variable that are used to aggregate the indicators to the one-dimensional index. The information on the infrastructural variable $p$ of household $i$, identified by $a_{ip}$, is a linear function of an unobserved factor, which in our case is the household's lack of infrastructure $c_i$. The relationship between the infrastructural variable $p$ in $c_i$ is given by $\beta_p$ plus a noise component $u_{ip}$, with both terms to be estimated from the data [3]. The estimation of the weights, which are used to aggregate the individual components of the index, a PCA as proposed by Filmer and Pritchett [2] for the construction of a wealth index is used to reduce the dimensions of the index. Then the first component, which compared to the later components includes the highest share of the original variation, is defined as the PDI. The PCA is structured by a set of equations where the household's infrastructural variable is related to a set of latent factors:

$$\begin{aligned} \tilde{a}_{1i} &= v_{11} A_{1i} + v_{12} A_{2i} + \ldots + v_{1p} A_{pi} \\ \ldots \\ \tilde{a}_{pi} &= v_{1p} A_{1i} + v_{p2} A_{2i} + \ldots + v_{pp} A_{pi}, \end{aligned} \tag{3}$$

where the $\tilde{a}$ are the $p$ infrastructural indicators (number of households that share a common toilet facility, public water source, people per room designated for sleeping, household has no bicycle, household has no motorbike or car, household has no mobile phone) normalized by their mean and their standard deviations. $\mathrm{A}_{pi}$ are the $p$ distinct principal components and $v$ are the weights (factor loadings) relating the principal component to the infrastructural variables similar as described in Filmer and Scott [4] for the construction of a household's wealth index based on the household's commodities. After the weights $v$ have been estimated, the inversion of the equation system shown in Equation 3 yields the following set of equations:

$$\begin{aligned} &A_{1i} = b_{11} \tilde{a}_{1i} + b_{12} \tilde{a}_{2i} + \ldots + b_{1p} \tilde{a}_{pi} \\ &\ldots \\ &A_{pi} = b_{1p} \tilde{a}_{1i} + b_{p2} \tilde{a}_{2i} + \ldots + b_{pp} \tilde{a}_{pi}, \end{aligned} \tag{4}$$

where the equation of the first principal component captures the highest share of the variation from the original set of variables. All other components are orthogonally aligned to the previous component (see also Fig. 1.7 for the amount of variance included in the first and succeeding components). The weights that are used to aggregate the variables into a one-dimensional index are given by the set $b_{11}, b_{21}, ..., b_{k1}$. The unweighted PDI is then calculated for each household. In the next step, the unweighted PDI is aggregated to the country and regional (admin-1) level, respectively using the household sample weight provided in the household member recode of the DHS. In the next step, considering that in more densely populated areas an infection with SARS-CoV-2 is more likely the unweighted PDI at the household-level, is aggregated to the regional (admin-1) and country-level by using $log(1 + \text{population density})$ as additional weight to achieve a weighting of the index. Pixel level estimates for the PDI are obtained by facilitating a Bayesian distributional regression approach and predicting the PDI for each pixel. See also Supplementary Materials 3.2 for details.

## 3.2 Bayesian distributional regression

**Distributional regression.** Identifying hotspots with a crucial lack of infrastructure, and accordingly, measures of physical distancing to slow down the spread of COVID-19 cannot be implemented effectively, are identified using Bayesian distributional regression models. This method can be characterized by the fact that all statistical distribution parameters can be related to a predictor. Since the distribution of any outcome is not described solely by its mean this is one important advantage to modeling the complete distribution. Ignoring this fact by applying standard regression models such as generalized linear models (GLMs), or generalized additive models (GAMs) can potentially yield conclusions drawn from a miss specified model [5].

Identifying hotspots with a high susceptibility for the transmission of COVID-19 caused by a lack of infrastructure is important to better target the allocation of scarce resources. The geo-spatial analysis takes advantage of Bayesian distributional regression models (that are also known as generalized additive models of location scale and shape [6]), to jointly relate all parameters of a given distribution to structured additive predictors [7]. Accordingly, all parameters of the underlying distribution of the PDI are related to a set of covariates that include the year of the survey, the population density of the primary sampling unit the household resides in, and the location of the primary sampling unit. As a starting point, the normal distribution for the PDI, $\text{PDI}_i \sim \mathcal{N}(\mu(x_i), \sigma(x_i)^2)$ is used since the PDI is unbound. Hence, for the normal distribution the regression model for the parameters $\mu$ and $\sigma$ is specified as follows:

$$\mu = h_1(\eta) = \text{id}(\eta_1), \sigma = \text{h}_2(\eta) = \exp(\eta_2), \tag{5}$$

where both parameters of the normal distribution are linked to their corresponding predictor $\eta_i$ through their appropriate response functions $h_i$, such that the restrictions of the parameter space are met. Thus the predictors $\eta_i$ for $\mu$ and $\sigma$ can be written as follows:

$$\eta_i = \beta_0 + f_1(\log(1 + \text{population density})) + \text{f}_2(\text{survey year}) + \text{f}_3(\text{longitude}, \text{latitude}), \tag{6}$$

which allows us to include to following model terms:

- Potentially non-linear effects of the population density and the survey year included in the effects $f_1(.)$ and $f_2(.)$.
- To account for spatial differences a spatially correlated effect $f_3$ of the location of the primary sampling unit is estimated as a two-dimensional surface. As pointed out by Lai et al. [8] spatial correlation over water has a different implication than over land, the models are estimated separately for sub-Saharan Africa and Madagascar.

For the estimation, Markov chain Monte Carlo (MCMC) simulation techniques are used and the estimation is carried out in the statistical software **R** (Version 3.5.1) [9] and the corresponding **R**-package **bamlss** (Version 1.1-3) [7, 10]. Specification of the statistical distribution is accomplished using the **R**-package **gamlss.dist** (Version 5.1-6) [11] that can be used for estimation in **bamlss**. Color palettes are based on the **R**-package **colorspace** (Version 2.0-0) [12, 13].

**Modelling approach.** The estimation approach is based on Bayesian distributional regression. It can be seen in Fig. 1.8 that the PDI is not symmetrical; accordingly, the assumed distribution needs to be able to account for the skewness. Furthermore, these figures highlight that the PDI is skewed and has heavier tails as one would assume from the normal distribution. Accordingly, the normal distribution is only chosen as a starting point. Then also, the t-distribution, the skew-normal distribution, and the skew t-distribution are used to give more weight to the tails and to account for skewness depicted in the histogram of Fig. 1.8. To compare the different models and to assess the models the deviance information criterion (DIC) [14], and the widely applicable information criterion (WAIC) [15] are used. See also Table 2.2. Both information criteria represent a trade-off between model fit and model complexity, with lower values of the DIC, respectively WAIC, indicating an improvement.

**Model diagnostic and check.** Besides the regression diagnostic based on the DIC and the WAIC (see Table 2.2, an addition, the fit of the distribution is graphically assessed by comparing the randomized quantile residuals [16]. See Fig. 1.9 for the corresponding plots of the final model. The fact that only minor deviations at the tails are visible indicates the chosen distribution of the final model fits reasonably well with the data. In-sample predictive performance is informally assessed by checking the probability integral transform (PIT)-histogram [17].

To assess in-sample predictive quality the probability integral transform (PIT)-histogram [17] is used to inspect if (in-sample) prediction quality. A uniform PIT-histogram indicates that the predictive distribution matches the distribution of the data reasonably. In the case of perfect prediction, the PIT-histogram would be uniform, a U-shape PIT-histogram would indicate under dispersion. See the corresponding panels of Fig. 1.9 that indicate a rather good match of the predictive distribution and the distribution of the data.

# Supplementary References


[1] Center for International Earth Science Information Network - CIESIN - Columbia University. Gridded Population of the World, Version 4 (GPWv4): Population Density, Revision 10. Data set, NASA SEDAC, Palisades, NY (2017). Figshare https://doi.org/10.7927/H4DZ068D.

[2] Filmer, D. & Pritchett, L. H. Estimating wealth effects without expenditure data—or tears: An application to educational enrollments in states of India. Demography **38**, 115–132 (2001). DOI: 10.1353/dem.2001.0003.

[3] Sahn, D. E. & Stifel, D. Exploring alternative measures of welfare in the absence of expenditure data. Review of Income and Wealth **49**, 463–489 (2003). DOI: 10.1111/j.0034-6586.2003.00100.x.

[4] Filmer, D. & Scott, K. Assessing asset indices. Working Paper 4605, World Bank, Washington, D.C. (2008). Preprint at https://doi.org/10.1596/1813-9450-4605.

[5] Umlauf, N. & Kneib, T. A primer on Bayesian distributional regression. Statistical Modelling **18**, 219–247 (2018). DOI: 10.1177/1471082X18759140.

[6] Rigby, R. A. & Stasinopoulos, D. M. Generalized additive models for location, scale and shape. Journal of the Royal Statistical Society: Series C (Applied Statistics) **54**, 507–554 (2005). DOI: j.1467-9876.2005.00510.x.

[7] Umlauf, N., Klein, N. & Zeileis, A. Bamlss: Bayesian additive models for location, scale, and shape (and beyond). Journal of Computational and Graphical Statistics **27**, 612–627 (2018). DOI: 10.1080/10618600.2017.1407325.

[8] Lai, Y.-S. et al. Spatial distribution of schistosomiasis and treatment needs in sub-Saharan Africa: a systematic review and geostatistical analysis. Lancet Infectious Diseases **15**, 927–940 (2015). DOI: 10.1016/S1473-3099(15)00066-3.

[9] R Core Team. R: a language and environment for statistical computing. R Foundation for Statistical Computing, Vienna, Austria (2021). https://www.R-project.org/.

[10] Umlauf, N., Klein, N., Simon, T. & Zeileis, A. bamlss: A lego toolbox for flexible Bayesian regression (and beyond). Journal of Statistical Software **100**, 1–53 (2021). DOI: 10.18637/jss.v100.i04.

[11] Stasinopoulos, M. & Rigby, R. gamlss.dist: distributions for generalized additive models for location scale and shape (2020). URL https://CRAN.R-project.org/package=gamlss.dist. R package version 5.1-6.

[12] Zeileis, A., Hornik, K. & Murrell, P. Escaping rgbland: Selecting colors for statistical graphics. Computational Statistics & Data Analysis **53**, 3259–3270 (2009). DOI: 10.1016/j.csda.2008.11.033.

[13] Zeileis, A. et al. colorspace: A toolbox for manipulating and assessing colors and palettes. Journal of Statistical Software **96**, 1–49 (2020). DOI: 10.18637/jss.v096.i01.

[14] Spiegelhalter, D. J., Best, N. G., Carlin, B. P. & Van der Linde, A. Bayesian measures of model complexity and fit. Journal of the Royal Statistical Society. Series B (Statistical Methodology) **64**, 583–639 (2002).

[15] Watanabe, S. Asymptotic equivalence of Bayes cross validation and widely applicable information criterion in singular learning theory. Journal of Machine Learning Research **11**, 3571–3594 (2010).

[16] Dunn, P. K. & Smyth, G. K. Randomized Quantile Residuals. Journal of Computational and Graphical Statistics **5**, 236–244 (1996). DOI: 10.1080/10618600.1996.10474708.

[17] Dawid, A. P. Present position and potential developments: some personal views: statistical theory: the prequential approach. Journal of the Royal Statistical Society. Series A (General) **147**, 278–292 (1984). DOI: 10.2307/2981683.
