# Supplementary material for: An index of access to essential infrastructure to identify where physical distancing is impossible
Source: Nat Commun. 2022 Jun 14;13:3355. doi: 10.1038/s41467-022-30812-8 (PMC9198068; doi:10.1038/s41467-022-30812-8)
Supplement: Supplementary file 3 — Reporting Summary [file 41467_2022_30812_MOESM3_ESM.pdf]

nature portfolio

Corresponding author(s): Dr. Kenneth Harttgen
DBPR
NCOMMS-21-38809A

Last updated by author(s): 2022/04/04

Reporting Summary

Nature Portfolio wishes to improve the reproducibility of the work that we publish. This form provides structure for consistency and transparency in reporting. For further information on Nature Portfolio policies, see our Editorial Policies and the Editorial Policy Checklist.

Statistics

For all statistical analyses, confirm that the following items are present in the figure legend, table legend, main text, or Methods section.

n/a	Confirmed	
☐	☒	The exact sample size (n) for each experimental group/condition, given as a discrete number and unit of measurement
☒	☐	A statement on whether measurements were taken from distinct samples or whether the same sample was measured repeatedly
☒	☐	The statistical test(s) used AND whether they are one- or two-sided<br>*Only common tests should be described solely by name; describe more complex techniques in the Methods section.*
☐	☒	A description of all covariates tested
☐	☒	A description of any assumptions or corrections, such as tests of normality and adjustment for multiple comparisons
☐	☒	A full description of the statistical parameters including central tendency (e.g. means) or other basic estimates (e.g. regression coefficient) AND variation (e.g. standard deviation) or associated estimates of uncertainty (e.g. confidence intervals)
☒	☐	For null hypothesis testing, the test statistic (e.g. F, t, r) with confidence intervals, effect sizes, degrees of freedom and P value noted<br>*Give P values as exact values whenever suitable.*
☐	☒	For Bayesian analysis, information on the choice of priors and Markov chain Monte Carlo settings
☒	☐	For hierarchical and complex designs, identification of the appropriate level for tests and full reporting of outcomes
☒	☐	Estimates of effect sizes (e.g. Cohen's d, Pearson's r), indicating how they were calculated

Our web collection on statistics for biologists contains articles on many of the points above.

Software and code

Policy information about availability of computer code

Data collection	The Demographic and Health Surveys (DHS) and the Gridded Population of the World (GPW) were downloaded from the DHS Program (https://dhsprogram.com/data/), and the Socioeconomic Data and Applications Center (https://sedac.ciesin.columbia.edu/data/collections/gpw-v4), respectively. Both data sets are public available third-party data.
Data analysis	For the estimation, Markov chain Monte Carlo (MCMC) simulation techniques are used and the estimation is carried out in the statistical software R (Version 3.5.1) and the corresponding R-package bamlss (Version 1.1-3). Specification of the statistical distribution is accomplished using the R-package gamlss.dist (Version 5.1-6) that can be used for estimation in bamlss. The color palettes are based on the R-package colorspace (Version 2.0-0).

For manuscripts utilizing custom algorithms or software that are central to the research but not yet described in published literature, software must be made available to editors and reviewers. We strongly encourage code deposition in a community repository (e.g. GitHub). See the Nature Portfolio guidelines for submitting code & software for further information.

Data

Policy information about availability of data

All manuscripts must include a data availability statement. This statement should provide the following information, where applicable:

- Accession codes, unique identifiers, or web links for publicly available datasets
- A description of any restrictions on data availability
- For clinical datasets or third party data, please ensure that the statement adheres to our policy

All data-sets used in this article are publicly available from the cited sources. The raw data consisting of the DHS data-sets and the Socioeconomic Data and Applications Center (SEDAC) gridded population of the world (GPW), v4 data-set, which are available from the following sources: DHS (https://dhsprogram.com/

data/); GPW (https://sedac.ciesin.columbia.edu/data/collection/gpw-v4). The underlying data which is based on the data-sets from the two previous sources and that support the results and conclusion drawn from this study is available upon reasonable request from the corresponding author.

Field-specific reporting

Please select the one below that is the best fit for your research. If you are not sure, read the appropriate sections before making your selection.

☐ Life sciences ☒ Behavioural & social sciences ☐ Ecological, evolutionary & environmental sciences

For a reference copy of the document with all sections, see nature.com/documents/nr-reporting-summary-flat.pdf

Behavioural & social sciences study design

All studies must disclose on these points even when the disclosure is negative.

Study description	This study used quantitative data and employs a mixed method approach. In particular, the study uses household surveys taken from the Demographic and Health Surveys and from the Socioeconomic Data and Applications Center. To calculate the measure of physical distancing, we apply a principle component analysis. In addition, we apply Bayesian simulation techniques to provide high resolution estimates for the index.
Research sample	Data were from the DHS, which are administered by ICF International and are nationally representative cross sectional surveys that have been conducted in over 82 low-and middle-income countries at varying intervals from 1985 and are still ongoing. The DHS are designed to collect nationally representative health and welfare data of women of reproductive age, their children, and their households. In addition, information on population density is taken from the Socioeconomic Data and Applications Center.
Sampling strategy	The DHS use a multi-stage stratified sampling design. Each country was divided into regions, which are political regions such as states/provinces or geographic areas divided and labeled north, south, east, and west. Within these sub-national regions, populations were stratified by urban and rural area of residence. Within these stratified areas, a random selection of enunmeration areas taken from the most recent population census was drawn. These primary sampling units were selected based on the probability proportional to the population size. In the second stage of sampling, all households within the cluster were listed and on average 25 houses within a cluster were randomly selected for an interview by equal probability systematic sampling. Detailed sampling plans are available from survey final reports. The DHS provided weights for calculating nationally representavive statistics.<br>The resulting sample is the largest data-set available at the household level.
Data collection	The DHS were downloaded from the DHS Program webpage (https://dhsprogram.com/data) and are publicly available. Information on population density is taken from the Socioeconomic Data and Applications Center and can be downloaded from the SEDAC webpage (https://sedac.ciesin.columbia.edu/data/collection/gpw-v4). This data-set is also publicly available.<br>For details on the data collection and sampling strategy see the DHS webpage (https://www.dhsprogram.com/Methodology/Survey-Types/DHS.cfm)
Timing	We employed the largest available, nationally representative, and mutually comparable repeated cross-sectional DHS data-set from sub-Saharan African countries for the most recent available survey year, which is between 2016 and 2018 for most countries, and no earlier than 2007. See the DHS webpage (https://www.dhsprogram.com/Methodology/Survey-Types/DHS-Methodology.cfm) for a detailed overview on the timeline and sampled countries.
Data exclusions	Only data with missing information has been excluded from the sample. No other data were excluded from the analysis.
Non-participation	Participating households were drawn in each enumeration area from a list of households within a specific enumeration area. See the DHS webpage (https://www.dhsprogram.com/Methodology/Survey-Types/DHS-Methodology.cfm) for details.
Randomization	Households are randomly displaced to guarantee their anonymity. DHS uses a two-stage sampling strategy. For details see: https://www.dhsprogram.com/methodology/Survey-Types/DHS-Methodology.cfm

Reporting for specific materials, systems and methods

We require information from authors about some types of materials, experimental systems and methods used in many studies. Here, indicate whether each material, system or method listed is relevant to your study. If you are not sure if a list item applies to your research, read the appropriate section before selecting a response.

Materials & experimental systems

n/a	Involved in the study	
☒	☐	Antibodies
☒	☐	Eukaryotic cell lines
☒	☐	Palaeontology and archaeology
☒	☐	Animals and other organisms
☒	☐	Human research participants
☒	☐	Clinical data
☒	☐	Dual use research of concern

Methods

n/a	Involved in the study	
☒	☐	ChIP-seq
☒	☐	Flow cytometry
☒	☐	MRI-based neuroimaging
